# Septic Arthritis of the Shoulder following Recent Varicella Zoster Virus Infection in an 18-month-old Child: A Case Report

**DOI:** 10.5704/MOJ.2511.020

**Published:** 2025-11

**Authors:** M Woodmass, LH Lee

**Affiliations:** Department of Trauma and Orthopaedic Surgery, Sunderland Royal Hospital, Sunderland, United Kingdom

**Keywords:** orthopaedics, paediatrics, shoulder, septic arthritis, varicella zoster virus

## Abstract

We present a case of an 18-month-old female child who attended the paediatric emergency department with mild dehydration, pyrexia and a healing chickenpox (varicella) rash. Her older brother recently recovered from chickenpox without complication and is believed to have been the source of her infection. Her classically self-limiting condition was initially treated supportively with oral fluids and benzydamine mouth spray, before two further presentations to the emergency department with worsening clinical deterioration, a protracted pyrexia and an exquisitely painful right shoulder prompted the search for an alternative diagnosis. This ordinarily fit and well, immunocompetent girl was subsequently diagnosed with septic arthritis of the right shoulder, complicated by concurrent varicella zoster virus infection. She was managed operatively with needle arthrocentesis, using a two-cannula technique to thoroughly wash out the infected glenohumeral joint. An intra-operative pus sample was sent for culture, subsequently growing Group A streptococcus pyogenes. Following washout, she received a course of intravenous antibiotics. There was immediate clinical recovery in the function of the right shoulder joint which continued into the post-operative period. Down-trending infection markers demonstrated a biochemical improvement, and she was discharged from hospital with a three-week course of oral antibiotics, making a full recovery. The rarity of upper limb involvement, along with the operative management technique of two needle arthrocentesis joint washout make this case an important contribution to the existing knowledge base and highlights the importance of considering this surgical emergency presenting in - what is usually - an overwhelmingly benign and self-limiting illness.

## Introduction

Varicella zoster virus (VZV) is the causative organism of varicella disease. It is more commonly known as chickenpox and is an extremely common infection in childhood, usually resulting in lifetime immunity^1^.

Overwhelmingly, paediatric chickenpox follows a benign clinical course. VZV is a highly contagious pathogen which can be transmitted via inhalation of respiratory droplets and by direct contact with blisters, saliva or mucus. Patients present with an acute fever followed by the development of a hallmark vesicular, blistering rash which may be intensely itchy. These blisters crust over to form scabs which heal over time. Management is supportive, focusing on symptomatic relief of itching and fever, along with oral fluids to maintain adequate hydration.

Complications are uncommon in the immunocompetent child and are usually limited to secondary bacterial soft tissue infections. Musculoskeletal complications in varicella are particularly rare, reportedly manifesting in just 0.01% of paediatric cases^2^. Here, we present an unusual case of a septic shoulder joint complicating concurrent varicella infection in an ordinarily fit and well 18-month-old girl.

This case is distinctive for two reasons. Firstly, its involvement of the glenohumeral joint is very unusual, as most cases of varicella arthritis affect the lower limb joints^3^. Secondly, we recount the use of an operative technique using two peripheral cannula needles to facilitate effective arthrocentesis washout of the septic joint.

## Case Report

An 18-month-old girl first presented to the paediatric emergency department with a 5-day history of malaise, reduced appetite, fever and a varicella rash. She is ordinarily fit and well, born at 38-weeks’ gestation with normal birthweight and developmental milestones. No known drug allergies were recorded. She lives with her parents and 4-year-old brother whom himself experienced an uncomplicated chickenpox rash 4 weeks ago. Neither parent had a history of cold sores or herpes simplex infection. She was discharged the same day with supportive care advice following review from the medical team.

Two days later, the family represented following concern expressed by her parents about a new finding: reluctance to use her right arm. Examination elicited generalised pain. No history of trauma or other physical injury was volunteered. Radiographs of the right shoulder and elbow demonstrated no evidence of bony injury, dislocation or a pulled elbow. She was again discharged with advice to continue supportive treatment and safety-netted to return if symptoms worsened or failed to improve.

The following day, the family presented for a third time. The girl appeared significantly more unwell, with drowsiness, rapid breathing and floppy tone. Examination revealed right-sided axillary lymphadenopathy and pain on moving the right upper limb at the shoulder joint. The shoulder itself was not erythematous but did feel warmer to the touch compared to the contralateral joint. Range of motion was very limited, and palpation caused significant distress to the child. This was an isolated joint involvement, with passive and active pain-free movement performed in all upper and lower limb joints, including the right elbow. Observations showed a pyrexia of 37.8° and a tachycardia of 172 beats per minute. There was growing concern of a septic arthritis due to the exquisitely painful shoulder joint and the unremitting pyrexia of 8 days’ duration.

**Fig. 1:**
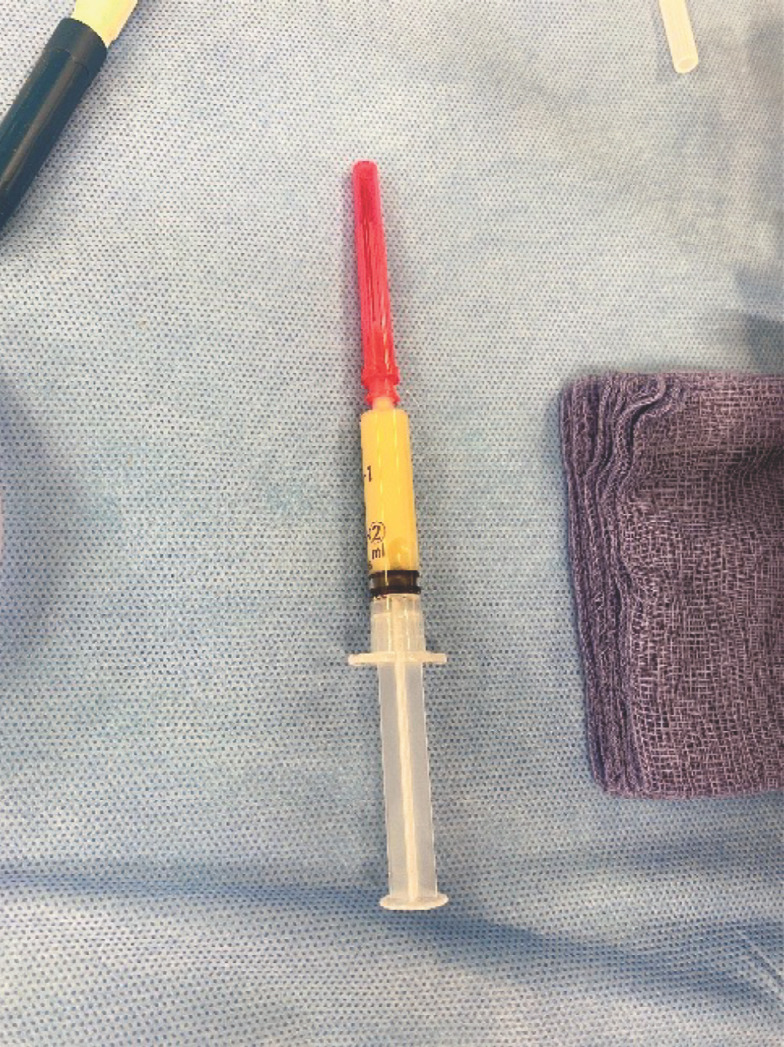
Aspirate contents from the infected glenohumeral joint, demonstrating purulent fluid.

**Fig. 2:**
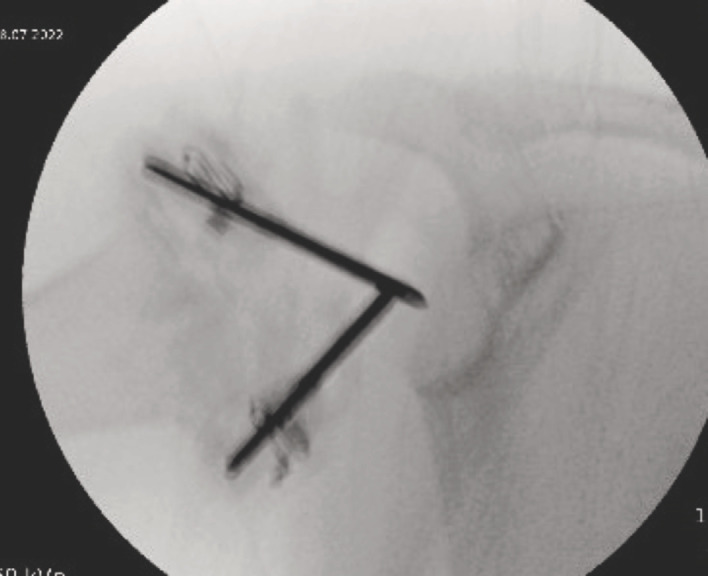
Intra-operative radiograph of arthrocentesis needle positions within the right glenohumeral joint.

**Fig. 3:**
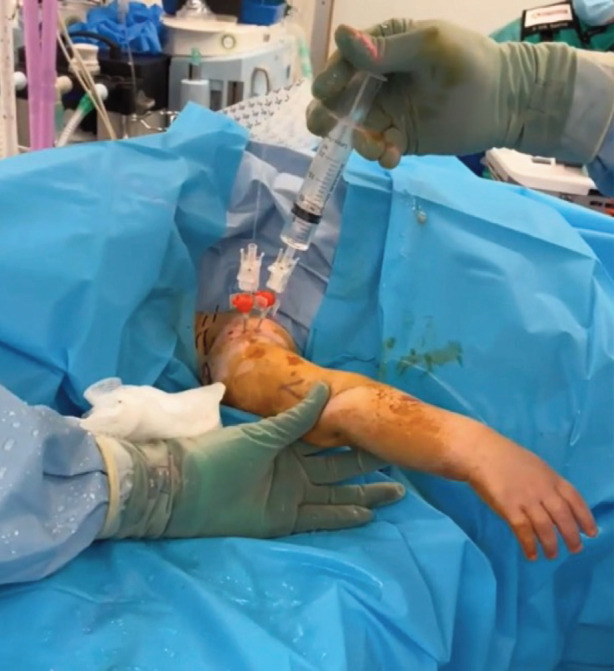
Irrigation and drainage of saline washout fluid via second cannula inserted into the right glenohumeral joint space.

The shoulder radiographs taken the previous day were re-reviewed and found no evidence of osteomyelitis or collection. A panel of blood tests demonstrated a significant bacterial infection (Table I). One stat dose of intravenous flucloxacillin was administered prior to the patient being taken to the operating theatre for aspiration of the right shoulder joint and needle washout. Under general anaesthesia, one 14-gauge peripheral intravenous cannula was inserted into the supraspinatus space, aspirating blood, and removed. Another 14-gauge cannula was inserted into the glenohumeral joint which yielded an aspirate of 2.5mls of purulent fluid (Fig. 1). An additional 14-gauge cannula was inserted to enable thorough wash out of the glenohumeral joint using one litre of warmed saline fluid (Fig. 2 and Fig. 3). An intra-operative pus sample was positive for Group A streptococcus pyogenes with sensitivities to flucloxacillin, amoxicillin, clarithromycin and cefalexin. Gram stain reported moderate white cells. The patient received intravenous flucloxacillin for five days total and converted to oral amoxicillin for a further three weeks.

**Table I:** Patient blood results with age-adjusted reference ranges.

Laboratory Test	Result	Reference Ranges - Age Adjusted
Hemoglobin (g/L)	95	115 – 140
Mean corpuscular volume (fl)	82.1	70 – 86
Hematocrit	0.289	0.34 – 0.40
White blood cells (x109/L)	30.82	6 – 17.5
Neutrophils (x109/L)	23.66	1.5 – 8.5
Lymphocytes (x109/L)	3.94	4.0 – 10.5
Platelets (x109/L)	670	150 – 400
Erythrocyte sedimentation rate (mm/Hour)	110	0 – 12
C-reactive protein (mg/L)	307.3	0 – 5

The patient was reviewed in clinic at three weeks with repeat radiographs, demonstrating a significantly improved range of motion of her right shoulder. Further clinic reviews at 6 weeks and 11 months noted no concerns and she was discharged thereafter.

## Discussion

Septic arthritis in paediatric patients necessitates joint drainage followed by administration of intravenous antibiotics^4^. There are several different methods of drainage. Arthrocentesis involves aspirating the infected joint with the insertion of a needle. A second option involves the insertion of an arthroscope. Alternatively, an arthrotomy may be considered which involves making a surgical incision to access the joint space. Needle aspiration is advantageous by being minimally invasive, carries a shorter procedure time and a quicker return to normal activity levels, but may require further procedures if there is inadequate clearance of pus^5^. Arthroscopy and arthrotomy are technically more demanding. Risks to the patient may include hemarthrosis and causing damage to surrounding cartilage, ligaments and nerves. Arthrotomy, carries the risk of significant scarring and post-operative joint stiffness. Consideration must also be paid to the small size of the patient which can preclude the use of bulky scopes and risky arthrotomy incisions. In our case, arthrocentesis was performed using an intravenous cannula which enabled effective irrigation to drain the pus from the joint space. An additional cannula was inserted into the glenohumeral joint to allow drainage of the saline fluid used for washout.

There is very limited available data on shoulder septic arthritis as a complication of varicella infection. This case serves to emphasise the importance to consider this as a possible diagnosis in patients who present with upper limb pain, particularly in the age group who cannot easily vocalise their symptoms or articulate the nature of their ailment.

The rapid deterioration of a varicella-infected child should prompt clinicians to consider this serious complication which, although rare, can have devastating consequences if not managed early. Musculoskeletal sequalae should not be overlooked and may necessitate surgical evaluation and intervention.
